# What makes a giant fruit? Assembling a genomic toolkit underlying various fruit traits of the mammoth group of *Cucurbita maxima*

**DOI:** 10.3389/fgene.2022.1005158

**Published:** 2022-09-20

**Authors:** Umesh K. Reddy, Purushothaman Natarajan, Venkata Lakshmi Abburi, Yan Tomason, Amnon Levi, Padma Nimmakayala

**Affiliations:** ^1^ Gus R. Douglass Institute and Department of Biology, West Virginia State University, Institute, Dunbar, WV, United States; ^2^ U.S. Vegetable Laboratory, USDA, ARS, Charleston, SC, United States

**Keywords:** cucurbit, mammoth, GWAS, sequencing, fruit, giant pumpkins

## Abstract

Since their introduction in Europe, pumpkins (*Cucurbita maxima* Duch.) have rapidly dispersed throughout the world. This is mainly because of their wide genetic diversity and Plasticity to thrive in a wide range of geographical regions across the world, their high nutritional value and suitability to integrate with local cuisines, and their long shelf life. Competition for growing the showy type or mammoth-sized pumpkins that produce the largest fruit of the entire plant kingdom has drawn attention. In this study, we used genome-wide single nucleotide polymorphisms to resolve admixture among different pumpkin groups. Also, to resolve population differentiation, genome-wide divergence and evolutionary forces underlying the evolution of mammoth-sized pumpkin. The admixture analysis indicates that the mammoth group (also called Display or Giant) evolved from the hubbard group with genome-wide introgressions from the buttercup group. We archived a set of private alleles underlying fruit development in mammoth group, and resolved haplotype level divergence involved in the evolutionary mechanisms. Our genome-wide association study identified three major allelic effects underlying various fruit-size genes in this study. For fruit weight, a missense variant in the homeobox-leucine zipper protein ATHB-20-like (S04_18528409) was significantly associated (false discovery rate = 0.000004) with fruit weight, while high allelic effect was consistent across the 3 years of the study. A cofactor (S08_217549) on chromosome 8 is strongly associated with fruit length, having superior allelic effect across the 3 years of this study. A missense variant (S10_4639871) on translocation protein SEC62 is a cofactor for fruit diameter. Several known molecular mechanisms are likely controlling giant fruit size, including endoreduplication, hormonal regulation, CLV-WUS signaling pathway, MADS-box family, and ubiquitin-proteasome pathway. This study provides a general framework for the evolutionary relationship among horticulture groups of *C. maxima* and elucidates the origins of rare variants contributing to the giant pumpkin fruit size.

## Introduction

Winter squash is the predominant domesticated form of *Cucurbita maxima* Duchesne. This species exists in South America, including Peru, Uruguay, Argentina, Bolivia, and Chile. Fossilized seeds, rinds, peduncles and even entire fruits of domesticated and weedy-type ancestral species (*C. andreana*) were excavated in many sites across the Ica Valley, Ocucaje, San Nicolas, and Chulpaca of Peru and sites located in south Chile and east Bolivia, with dating up to 7000 B.C-A.D.1750 (Cutler and Whitaker, 1961; Nee, 1990; Sanjur et al., 2002). Kates et al. (2017) resolved the phylogenetic and domestication history and placed *C. maxima* along its ancestral wild species *C. ecuadorensis* clade as basal to the rest of the North American mesophytic forms. Adaptation of *C. maxima* to the wetter climates and evolution of the mesophytic habitat was probably the basis for the diversification and distribution of the rest of the *Cucurbita* species across the American continent. Kates et al. (2017) further resolved the *C. maxima* clade into the wild and domesticated sister clade, with signatures of reduced heterozygosity while domestication, and further elucidated domestication bottlenecks in the evolutionary history.

In the La Plata lowlands of the Amazon basin, widespread evidence of domestication of pumpkin associated with the Guarani and Arawak cultures was unearthed in mounds across southeastern Argentina and Uruguay (Bonomo et al., 2021). The fruit types of *C. maxima* include Hubbard, Nugget, Banana, Giant pumpkin, Buttercup, and Turban, with diverse exterior features. In addition, there are wide-ranging bush types with small-sized fruits similar to the Zapallito type that can be consumed as summer squash (Tapley et al., 1937; Culpepper and Moon, 1945; Cutler and Whitaker, 1961; Nee, 1990; Ferriol et al., 2004; Ferriol and Picó, 2008; López-Anido, 2021). In *C. maxima*, peduncles and seeds are the best diagnostic to identify various horticulture groups (Whitaker and Carter, 1946). Seeds are white in *C. maxima* and brown in the wild sister *C. andreana*, with flat or oval shapes. Seeds are 10–30 mm in length and 5–20 mm wide in cultivated *C. maxima* but 5–10 mm long and 4–7 mm wide in *C. andreana*. The diameter of peduncles of *C. maxima* is up to 15 mm but 8 mm for *C. andreana*. Fruit shape varies from banana to drum-shaped, top-shaped, fusiform, oblate with rind colors ranging from red-orange to pink-orange, nearly white, bluish gray, intense green, and black-green (López-Anido, 2021).

The Goldman (2004) classification as Australian, Hubbard, Buttercup, Banana, Turban, Mammoth, and Zapallito cultivar groups is currently widely adopted (Kazmińska et al., 2016; López-Anido, 2021). Since their introduction during the early 16th century in Europe, pumpkins rapidly dispersed across the world mainly because of their suitability to become integrated in the local cuisine; diverse nutritive values, possessing sugars, vitamins, carotenoids, higher fiber content, and seed fatty acid profiles; coupled with a long shelf life and wide adaptation (Esquinas-Alcazar and Gulick, 1983; Kazmińska et al., 2016). *C. maxima* then underwent a great diversification in their China–Japan and India–Myanmar secondary domestication centers, respectively (Nee, 1990; Ferriol and Picó, 2008). The supreme mature fruit-flesh quality of *C. maxima* might have played a major role in rapid population expansion soon after its introduction in Europe during the early 16th century. Breeding programs across the world soon admixed various groups to produce a diverse Australian group such as a drum-shaped and dark green or black-green kabocha, a high quality winter melon in Japan that sharply increased the consumption of pumpkins and winter squash (Ratnayake et al., 2004). An iconic cultivar, *C. maxima* ‘Buttercup’, which was bred at the North Dakota Agricultural Experiment Station (Yeager and Latzke, 1932), is today the standard by which all the other pumpkins and winter squash are rated for quality. Balkaya et al. (2010) studied the morphological diversity of various horticultural groups from Turkey and noted wide variance in fruit weight, fruit diameter, fruit length, length of seed cavity, and flesh thickness. The current study focused on the admixture of various lineages within *C. maxima* to form diverse horticulture groups.

The fruit size in the mammoth type is apparently an interaction of physiological sink and the genetic control underlying cell size and cell number (Janick, 2008). Large-fruited pumpkin have a more extended period of cell division and greater cell expansion after cell division ceases than fruits of smaller cucurbits (Sinnott, 1939). In the larger showy pumpkins, the period from seeding to harvest is about 130–140 days, whereas the period from pollination to harvest is about 60–80 days, which may be under genetic control (Janick, 2008). Kaźmińska et al. (2017) performed genome-wide analysis using 23 simple sequence repeat markers to separate the mammoth-sized horticulture group as a separate cluster from the rest of the *C. maxima* types.

The objective of this study is to elucidate the genome-wide molecular diversity and evolution in a representative collection involving major groups of *C. maxima*, and determine genetic factors underlying the growth and development of the giant pumpkins (Guinness World Records, 2021) (Pan et al., 2022). It aimed to reveal the genetic components of the mammoth horticulture group with reference to the other horticulture groups and explore the possible genetic factors underlying the giant fruit growth. It further aimed to develop informative single nucleotide polymorphism (SNP) markers associated with fruit traits that contributed to the fruit size.

## Materials and methods

### Plant material and growth conditions

A collection of 100 heirlooms of *C. maxima* belonging to the cultivar groups Australia, Hubbard, Buttercup, Banana, Turban, Mammoth, and Zapallito (Supplementary Table S1; Supplementary Figure S1) were grown in a 10-m^2^ plant area and evaluated under field conditions during the years 2010, 2011, and 2012, adopting three replications, each replication consisting of 10 plants per accession.

### Fruit morphology measurements

Fruit weight (FW; kg), fruit length (cm) (Anastasiou et al.), fruit diameter (FD; cm), ratio of fruit length and fruit diameter (RLD; %), and soluble solids (SOL; μg/g) were observed for five individual plants at maturity.

### Genotyping-by-sequencing, mapping reads to the reference genome and SNP calling

The seedlings were collected, and genomic DNA was extracted by using the DNeasy plant mini kit (QIAGEN, Germany). Samples (DNA plus adapters) were digested with the restriction enzyme *ApeK*I, a type II restriction endonuclease, and Illumina HiSeq 2500 was used for sequencing as described (Elshire et al., 2011). The GBS reads from *C. maxima* genotypes were mapped to the *C. maxima* reference genome (http://cucurbitgenomics.org/organism/8) by using the Burrows-Wheeler Aligner tool (http://bio-bwa.sourceforge.net/). The mapped GBS reads were used to call SNPs with the GB-eaSy tool (https://github.com/dpwickland/GB-easy). The resulting variant call file (vcf) was used for further downstream analysis.

### Population structure analysis of *C*. *maxima* accessions

A total of 47,568 SNPs were filtered by minor allele frequency (MAF) = 0.05 and call rate 70% to identify 12,996 SNPs. Structure analysis of the SNPs involved using Structure v.2.3.4 (https://web.stanford.edu/group/pritchardlab/structure.html). The population structure was constructed with the following parameters: 1) length of burning period: 50,000, 2) number of Markov Chain Monte Carlo reps: 100,000, 3) K used: 1-8 and 4) number of replication runs: 3. Structure harvester (http://taylor0.biology.ucla.edu/structureHarvester/) was used to identify the optimal K based on the DeltaK value. To analyze population structure, we used 12,996 SNPs of *C*. *maxima* for principal component analysis (PCA). Genotype positions in PCA were color-coded according to cultivar groups. The eigenvalues were estimated by using the EIGENSTRAT algorithm with SNP & Variation Suite (SVS v8.1.5) (Golden Helix, Inc., Bozeman, MT, United States; www.goldenhelix.com) (Patterson et al., 2006).

### Genes under selection

To identify candidate genes involved in mammoth fruit generation, we combined three approaches to select positive selection genes (PSGs), including intergroup differentiation index (*F*_ST_), nucleotide diversity ratios (*π*_buttercup_/*π*_mammoth_ and *π*_*hubbard*_/*π*_*mammoth*_) of the mammoth group to buttercup and hubbard separately and Tajima’s *D* within the mammoth group. We calculated all diversity indices (*F*_ST_, *π*, and Tajima’s *D*) with a 2-kb sliding window in VCFtools (–fst-window-size 2000 –weir-fst-pop, –Tajima’sD 2000, –window-pi 2000). If sites were under strong positive or purifying selection in the mammoth group, a relatively high genetic divergence and a decrease in genetic diversity was expected as compared with the buttercup or hubbard population. The windows exhibiting extremely high values of *F*_ST_ and *π*_buttercup_/*π*_mammoth_ and *π*_*hubbard*_/*π*_mammoth_ (using the top 5% quantile of the simulated distribution), and the negative Tajima’s *D* were selected as PSGs.

### Association mapping

Mapped SNPs obtained from GBS data were used to prevent spurious linkage disequilibrium (LD) and thus unreliable association mapping. SVS v8.1.5 (Goldenhelix Inc.) was used for genome-wide association study (GWAS) by adopting multiple-locus mixed linear models developed by using the Efficient Mixed-Model Association eXpedited (EMMAX) method and implemented in SVS v8.1.5. For GWAS, the PC matrix and identity-by-descent indices were used as covariates to reduce the confounding effects of population substructure and kinship. Manhattan plots for associated SNPs were visualized in GenomeBrowse v1.0 (Golden Helix, Inc). Associated SNP *p*-values from GWAS were analyzed by false discovery rate (FDR). A total of 12,996 SNPs for *C*. *maxima* were used in GWAS to identify alleles that affect various fruit traits.

### Annotation

A program for annotating and predicting the effects of SNPs, SnpEff, was used to analyze PSG locations and associated SNPs underlying giant fruit size (Cingolani, 2022). SnpEff annotates variants based on their genomic locations and predicts coding effects. Annotated genomic locations include intronic and untranslated regions, upstream and downstream regions, splice site, or intergenic regions. Coding effects such as synonymous or non-synonymous amino acid replacement, start codon gains or losses, stop codon gains or losses, or frame shifts can be predicted.

## Results

### SNP development, haplotype distribution and linkage disequilibrium decay

A total of 12,996 SNPs (MAF ≥0.05) were isolated from the nucleotide sequences obtained for the 100 *C*. *maxima* accessions. The SNPs 873, 759, 670, 1266, 605, 624, 553, 498, 577, 512, 853, 634, 475, 864, 593, 577, 535, 628, 456, and 444 were mapped to the *C*. *maxima* reference genome and were located on chromosomes 1, 2, 3, 4, 5, 6, 7, 8, 9, 10, 11, 12, 13, 14, 15, 16, 17, 18, 19, and 20, respectively. A total of 1102 SNPs were in 488 haplotypes across various chromosomes at 90% call rate. The haplotypes were 30, 19, 13, 52, 27, 25, 14, 23, 16, 12, 34, 27, 14, 41, 26, 22, 21, 27, 11, and 16 and were located on chromosomes 1, 2, 3, 4, 5, 6, 7, 8, 9, 10, 11, 12, 13, 14, 15, 16, 17, 18, 19, and 20, respectively. Average LD sizes were 2.41, 0.97, 1.64, 3.45, 1.05, 2.78, 2.22, 3.00, 3.38, 3.02, 1.90, 2.95, 3.07, 2.24, 3.30, 2.05, 2.45, 2.41, 1.49, and 2.64 and were located on chromosomes 1, 2, 3, 4, 5, 6, 7, 8, 9, 10, 11, 12, 13, 14, 15, 16, 17, 18, 19, and 20, respectively. The largest LD block sizes were 52.64, 21.50, 33.69, 128.85, 22.96, 73.04, 17.61, 40.46, 58.35, 38.77, 45.79, 45.78, 42.76, 32.94, 58.78, 48.36, 71.56, 67.19, 26.42, and 34.14 and were located on chromosomes 1, 2, 3, 4, 5, 6, 7, 8, 9, 10, 11, 12, 13, 14, 15, 16, 17, 18, 19, and 20, respectively.

## Morphological variation

Cultivar groups in this study (Supplementary Figure S2 ; Supplementary Table S2) showed wide variation for various quantitative traits: mammoth had the largest fruit size and Zapallito, the smallest. In this study, the weight of mammoth mature fruit was about 8–10 kg, which is much smaller than their standard size of 100–600 kg because all accessions belonging to various horticulture groups were grown in a small planting area of 10 m^2^. In addition, the experiment was performed during the summer, under high temperatures which limited the entire plant and fruit growth and development. A uniform plot size was adopted for all horticulture groups for comparing various traits in this study. All mammoth types (Atlantic Giant, Big Max, Wyatt’s Wondef, Atlantic, Narodnaya, Volgskaia Seraia, Dill’s Atlantic Giant, Full moon, Big moon, Mamont, Stofunt, Amish Pie, King of Mammoth ,and Russian kit) had large, round fruits with orange and yellow flesh and diverse color exteriors. The horticulture groups featured striking differences. The hubbard group (Golden Hubbard, Chicago Warted Hubbard, Hubbard True Green, Baby Green Hubbard, Red Hubbard, Blue Hubbard, and Boston Marrow) were semi-bush type plants with fruits of characteristic elliptical or ovate shape and tapered ends; the exterior contained rough warts. Some of the characteristic buttercup types were Rouge vif d’Etampes, Confection, Bush Buttercup, Golden Hound, Baby Delica, Blue Kuri, Naguri, Red Warty Thing, Kabocha, Blue Doll, Ulibka, Bush Buttercup, Burgress Buttercup, Sviten, Marmellata, Bonbon, Tronco, Blue Doll, Lower Salmon River and N22 and were the most diverse type fruits, with greenish rinds, and sweetish flavored flesh. Turks Cup and Mini Red Turban were the only turban types in the study and had a relatively vine growth habit with smaller leaves. Turban fruits were small with orange- or yellow-colored flesh. The banana types were Jumbo Pink Banana, Candy Roaster, Sibley, Guatemalan Blue, Swedish Banana and Pastila Champang, and were characteristic, with elongated and locular fruits with pinkish exteriors. Zapallito was the small pumpkin type and the consumption is largely summer squash. The Australian types are more or less improved buttercup types or largely admixed with the hubbard types, and raw fruits are flat with diverse colored rinds.

### Admixture of various cultivar groups of *C. maxima*

**FIGURE 1:**
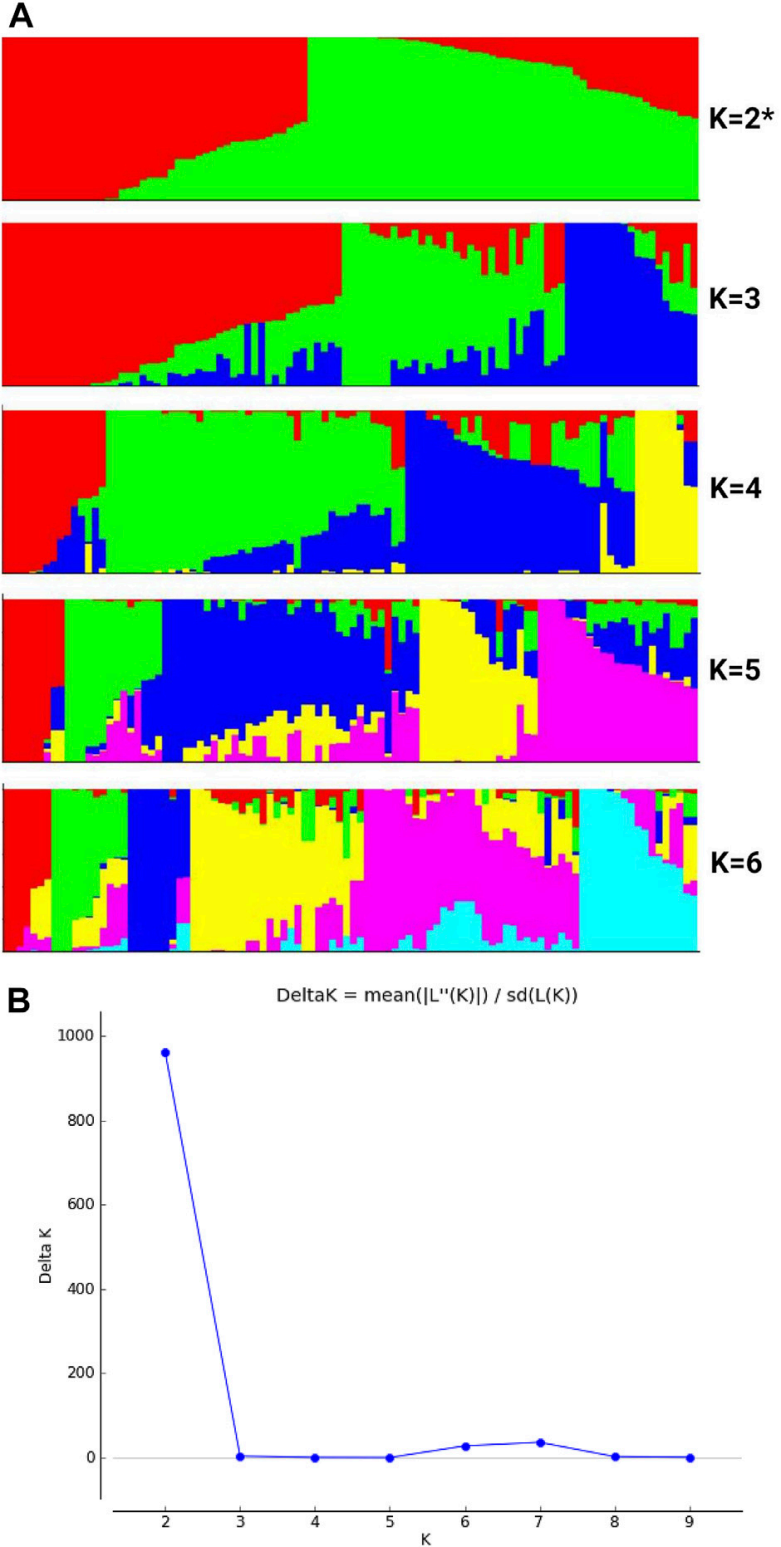
a and 1b: **(A)** Estimated population structure of 100 accessions of *Cucurbita maxima* on *K* = 6. *Accessions in red are clustered into pop1 and those in green are clustered into pop2*. **(B)** Delta *K* (Δ*K*) for different numbers of subpopulations (*K*).

The main objective of this study was to resolve admixture among various horticulture groups of *C. maxima* and to resolve core collections of various cultivar groups in this study. We conducted population structure analysis, a model-based admixture analysis, with the resulting dataset for 100 accessions and 12,996 SNP markers by using Structure v.2.3.4 to resolve independent lineages (Figure 1A). The analysis is based on model-based assumptions, and the structure algorithm is based on a Bayesian approach for choosing the number of clusters to be formed. Delta K values were estimated by using Structure Harvester. We tested the population structure for *K* = 2 to 6 with 3 iterations and based on the significance of Delta K values; the appropriate number of clusters was 2 for our dataset (Figure 1B). Population structure analysis showed two clusters (in red and green colors) for buttercup, Australian, zapallito and turban types as one group (red) and hubbard and banana types as the other group (green). The red lineage contained Baby Delica, Chihuahua, Rouge vif d’Etampes, Confection, Golden Hound, Buttercup, Delica, Blue kuri, Naguri, N21, Red Warty Thing, Kabocha, Bush Buttercup, Hokkaido, Ulibka, Blue Doll, Khersonkaia, Ubileinaya, Khersonskaia, Burgress Buttercup, Argentina Khersonskaia, primitive, Sviten, Marmellata, Burgress, Cha-Cha, Turks Cup, Mini Red Turban, Sri Tong and Zappallito De Tronco, with no admixture of the green lineage. This lineage was predominant in buttercup types, Australian types, zapallito, and turbon types. The second lineage was predominantly hubbard, mammoth and banana types (N22, Sweedish, Banana, Chihuahua, Blue Hubbard, Golden Habbard, Rossianka, Queensland, Blue, N19, Queen, Burgress, Flat white and Boer) containing fruits elliptical or ovate in shape with tapered ends, or elongated and locular fruits. All the giant size mammoth pumpkins were admixed in K-2 with the majority the hubbard type lineage and 2%–45% the buttercup type lineage (Atlantic giant: 0.15 buttercup: 0.85 hubbard); Silver moon (0.13 buttercup: 0.87 hubbard); N6 (0.13 buttercup: 0.87 hubbard); Mammoth (0.09 buttercup: 0.91 hubbard); Kit (0.09 buttercup: 0.91 hubbard); Full moon (0.07 buttercup: 0.93 hubbard); Big moon (0.04 hubbard: 0.96 buttercup); Big max (0.02 hubbard: 0.98 buttercup); Amish Pie (0.07 buttercup: 0.93 hubbard); Mamont (0.25 buttercup: 0.75 hubbard); Wyatt’s Wonder (0.45 buttercup: 0.55 of hubbard); Narodnaya (0.35 buttercup: 0.66 hubbard); Stofunt (0.34 buttercup: 0.66 hubbard); and Volgskaia seraria (0.19 buttercup: 0.81 hubbard), which indicates that mammoth is predominantly a hubbard type with a moderate size admixture of buttercup lineage. However, in the K-3 analysis of population structure, the mammoth type formed a separate group, and the K-4 and K-5 analysis resolved the rest of the world collections including flat types and round types of the Australian group. The Australian group is a highly admixed group consisting mostly of improved heirlooms. We performed a principal component analysis (PCA) involving all the accessions belonging to various groups and a PCA chart was made using first two eigen vectors. PCA in this study, indicated three overlapping clusters involving 1. buttercup, zapallito and turbon 2. hubbard and banana and 3. mammoth types along with few admixed genotypes (Supplementary Figure S3).

## Distribution of genetic diversity

**FIGURE 2:**
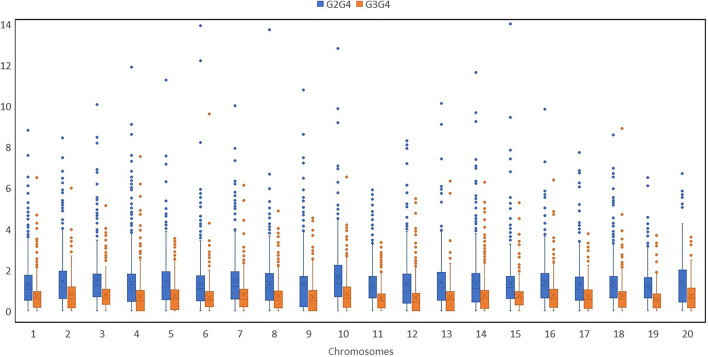
Box plots of various chromosomes depicting nucleotide diversity ratios (*π*_buttercup_/*π*_*mammoth*_ and *π*_*hubbard*_/*π*_*mammoth*_) of the buttercup to hubbard and mammoth groups.

We noted higher nucleotide diversity across various chromosomes for buttercup, turban and zapallito types (group 2) and hubbard plus banana types (group 3) as compared with the mammoth type (group 4) (Table 1). Nucleotide diversity (π) was estimated for various groups by using filtered SNPs to estimate the ratios of nucleotide diversity of buttercup to mammoth and hubbard to mammoth (Figure 2). This allowed for tracking how genetic diversity contributed to the formation of the mammoth group and location of positive selection genes across the chromosomes from the two ancestral horticulture groups. Ratios of nucleotide diversity (*π*_buttercup_/*π*_mammoth_ and *π*_*hubbard*_/*π*_mammoth_) further revealed that the mammoth group is close to the hubbard rather than buttercup group.

**TABLE 1:** Chromosome-wide distribution of nucleotide diversity (π) across *Cucurbita maxima* groups.

	Australian	Buttercup	Hubbard	Mammoth
Mean for Chromosome 1	3.57352E-05	4.29328E-05	4.29765E-05	3.74968E-05
2	3.80693E-05	4.3798E-05	4.47186E-05	3.59468E-05
3	3.68298E-05	4.59182E-05	4.63991E-05	3.83997E-05
4	3.52784E-05	4.13103E-05	4.31357E-05	3.47611E-05
5	3.46217E-05	4.31369E-05	4.38399E-05	3.82114E-05
6	3.48301E-05	4.11271E-05	4.12778E-05	3.59462E-05
7	3.58517E-05	4.372E-05	4.57546E-05	3.58532E-05
8	3.31093E-05	4.09917E-05	4.25236E-05	3.50819E-05
9	3.46721E-05	4.46094E-05	4.47175E-05	3.75746E-05
10	3.51923E-05	4.49279E-05	4.4724E-05	3.29029E-05
11	3.58378E-05	4.42008E-05	4.27045E-05	3.79088E-05
12	3.56228E-05	4.19834E-05	4.06528E-05	3.64941E-05
13	3.36346E-05	4.236E-05	4.37332E-05	3.74815E-05
14	3.52168E-05	4.0485E-05	4.14544E-05	3.31868E-05
15	3.41631E-05	4.27887E-05	4.33182E-05	3.74491E-05
16	3.4425E-05	4.31859E-05	4.35216E-05	3.75117E-05
17	3.17415E-05	3.81267E-05	4.12052E-05	3.3657E-05
18	3.65E-05	4.97328E-05	4.49518E-05	3.73295E-05
19	3.56284E-05	4.09151E-05	4.1563E-05	3.82255E-05
20	3.32473E-05	4.33644E-05	4.30689E-05	3.15856E-05
Grand mean	**3.50104E-05**	**4.29808E-05**	**4.3312E-05**	**3.61502E-05**

## Private alleles specific to mammoth

In addition, we tracked 32 private alleles specific to the mammoth type that were specific to this group. These private alleles were spread across chro-1 (1), chro-2 (4), chro-3 (4), chro-4 (4), chro-5 (1), chro-7 (1), chro-8 (3), chro-9 (1), chro-11 (3), chro-12 (2), chro-13 (1), chro-14 (3), chro-15 (1) and chro-18 (2). Some of the genes harboring mammoth-specific private alleles were in S-acyltransferase, asparagine synthetase, ubiquitin carboxyl-terminal hydrolase, WRKY, RING-type E3 ubiquitin transferase, cell division protein ftsZ, trypsin family protein, Ferredoxin, (1->3)-beta-glucan endohydrolase, -hydroxyacyl-CoA dehydrogenase, protein RRP6-like 2, delta (24)-sterol reductase, C2 calcium/lipid-binding plant phosphoribosyltransferase family protein, choline-phosphate cytidylyltransferase, SAM domain-containing protein, tetraspanin-8, FIP1, rhodanese domain-containing protein, cytochrome c oxidase subunit 6b-1-like, skin secretory protein xP2-like isoform X1, mediator of RNA polymerase II transcription subunit 8 isoform X1, hydroxyproline-rich glycoprotein, and pectin lyase-like superfamily protein.

## Genome-wide divergence and signatures of a selection of the mammoth group

**FIGURE 3:**
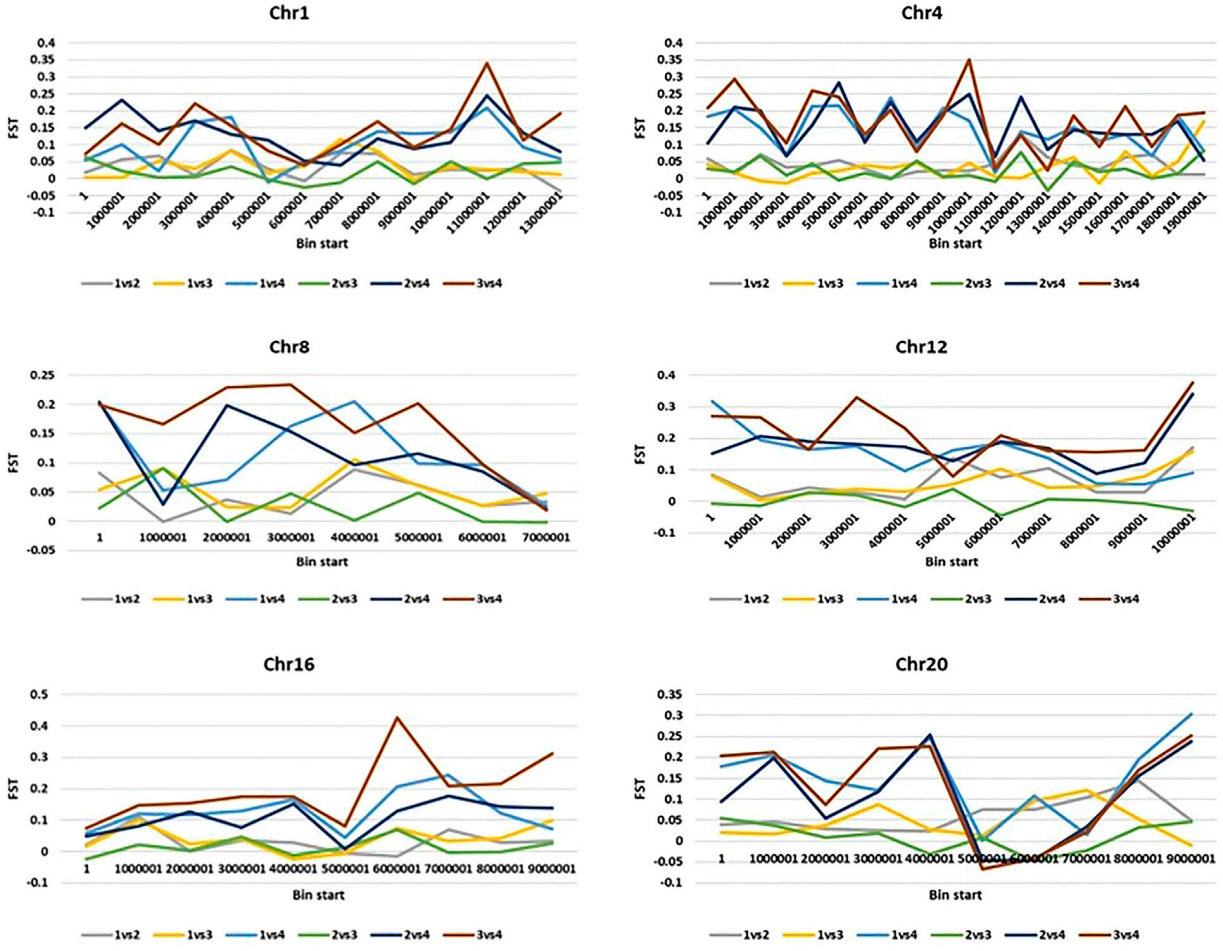
2-kb sliding windows of chromosomes 1,4,8,12,16 and 20 depicting pairwise *F*_*ST*_s of various groups (I: Australian, II: Buttercup, Zapallito, Turban, III: Hubbard and Banana and IV: Mammoth).

We further calculated pairwise *F*_*ST*_ with a 2-kb sliding window by using filtered SNPs in VCFtools to show genomic locations with maximum and least divergence occurring genome-wide with respect to mammoth types and the other horticulture groups (Figure 3 and Supplementary Figure S4). We used genome-wide estimates of Tajima’s D values to investigate selection in the site frequency spectrum that could result from mammoth group evolution, with negative values indicating excess rare variants due to purifying selection and positive values indicating excess intermediate frequency variants due to positive selection. The top (positive) and bottom 5% (negative) quantile of the simulated distribution of Tajima’s D indices across various chromosomes widely differed for the mammoth group and the other groups, so this group formation was recent and underwent genome-wide changes (Supplementary Figure S5). Genes underlying positive and negative Tajima’s D were subjected to Gene Ontology (GO) analysis to understand selection within the cellular location, molecular function, and biological process in the mammoth group (Supplementary Figure S6). GO analysis provided a uniform vocabulary for specifying cellular location, molecular function, and biological process. We generated ontologies separately for the top 5% quantile of the simulated distribution intergroup differentiation index and nucleotide diversity ratios and Tajima’s D indices and compared these to note the same vocabulary of cellular location, molecular function, and biological process. The biological processes under selection in the mammoth group formation were organic substance metabolic process, primary metabolic process, nitrogen compound metabolic process and transmembrane transport; the molecular functions related to transferase activity, hydrolase activity, heterocyclic compound binding, organic cyclic compound binding, transferase activity and catalytic activity. The main cellular locations at which the genes under selection function were organelle, intracellular anatomical structure and membrane transport systems. The genes with multiple SNPs and positive Tajima’s D were elastin-like and MYB-related protein 305-like on chro-4; DExH-box ATP-dependent RNA helicase DExH5, WRKY transcription factor 13 and transcriptional regulator SLK2 on chro-18; Protein of unknown function (DUF3537) on chro-5; YTH domain-containing protein and pectinesterase on chro-17; glycerol-3-phosphate acyltransferase RAM2 and Protein DJ-1-like protein D-like on chro-14; Chaperonin 60 subunit beta 1, chloroplastic on chro-11; zinc_ribbon_12 domain-containing protein and cullin-associated NEDD8-dissociated protein 1 on chro-16; E3 ubiquitin-protein ligase Praja-2 and protein indeterminate-domain 2-like on chro-3; transcription factor MYB4-like, ATP-dependent DNA helicase and autophagy-related protein 9 on chro-19; vacuolar cation/proton exchanger and serine/threonine receptor-like kinase NFP on chro-2; GDAP2 homolog and pentatricopeptide repeat-containing protein on chro-12; berberine bridge enzyme-like 26 and serine/arginine-rich splicing factor RSZ22A on chro-1; RING-type domain-containing protein on chro-13; ethylene-responsive transcription factor ERF020 on chro-15; polyphenol oxidase, chloroplastic-like on chro-13; and chloride channel protein on chro-6. Similarly, genes harboring multiple SNPs with negative Tajima’s D were DUF4408 domain-containing protein in chro-2; protein PHOX1-like and H (+)-exporting diphosphatase on chro-10; factor of DNA methylation 4, cationic amino acid transporter 1-like and high-affinity nitrate transporter 2.2 on chro-13; cell number regulator 6-like, separase, calcium-dependent ARF-type GTPase activating protein family and GDSL esterase/lipase on chro-4; FAM186A-like on chro-8; MLP-like protein 34 and pectinesterase on chro-14; Branched-chain-amino-acid aminotransferase and chromatin-remodeling ATPase INO80 on chro-3; mitogen-activated protein kinase 12-like isoform X1 on chro-15; methyltransferase subunit TRM112-like protein on chro-17; GATA transcription factor on chro-18; mitochondrial carrier protein on chro-1; ethylene-responsive transcription factor 2-like on chro-16; and enoyl-[acyl-carrier-protein] reductase [NADH], and chloroplastic-like on chro-20. SNPs located in these genes caused selective sweeps across various chromosomes and hereafter referred as positive selection genes (PSGs) (Supplementary Tables S3 and S4).

## Common genes under selection

From the top 5% quantile of the simulated distribution of intergroup differentiation index (*F*_ST_) and nucleotide diversity ratios, we listed candidate genes and compared these genes with Tajima’s D values to identify common genes under selection in both analyses. We compiled a final set of genes that was common for all the three approaches and annotated their function. The genes in common with largest intergroup differentiation index and nucleotide diversity ratios with positive Tajima’s D indices (positive selection) were alpha/beta-Hydrolases superfamily protein cell number regulator 6-like, DUF641 domain-containing protein, E3 ubiquitin-protein ligase COP1-like, pentatricopeptide repeat-containing protein, serine/threonine phosphatase, RING-H2 finger protein ATL34-like, transcription factor MYB36-like, WRKY protein and WD-40 repeat-containing protein. Positive selection signifies a decrease in population size coupled with balancing selection. In contrast, genes harboring SNPs with high *F*_*ST*_ and high nucleotide diversity but purifying selection with negative Tajima’s D value were calcium-dependent protein kinase-like, auxin-responsive protein, cyclic nucleotide gated channel, GDSL esterase/lipase, mitogen-activated protein kinase, and subtilisin-like protease SBT2.5, meaning an excess of low-frequency polymorphisms due to bottlenecks in mammoth group formation and expansion of population size. Pectinesterase and ethylene-responsive transcription factor 2-like were also important selection genes with partly negative and positive selective sweeps indicating intragenic evolution (Supplementary Tables S3 and S4). Overall Tajima’s D values for entire set of accessions in this study is presented in Supplementary Table S5; Supplementary Figure S7.

### Haplotype sharing, gain and loss of haplotypes across the genome

**FIGURE 4:**
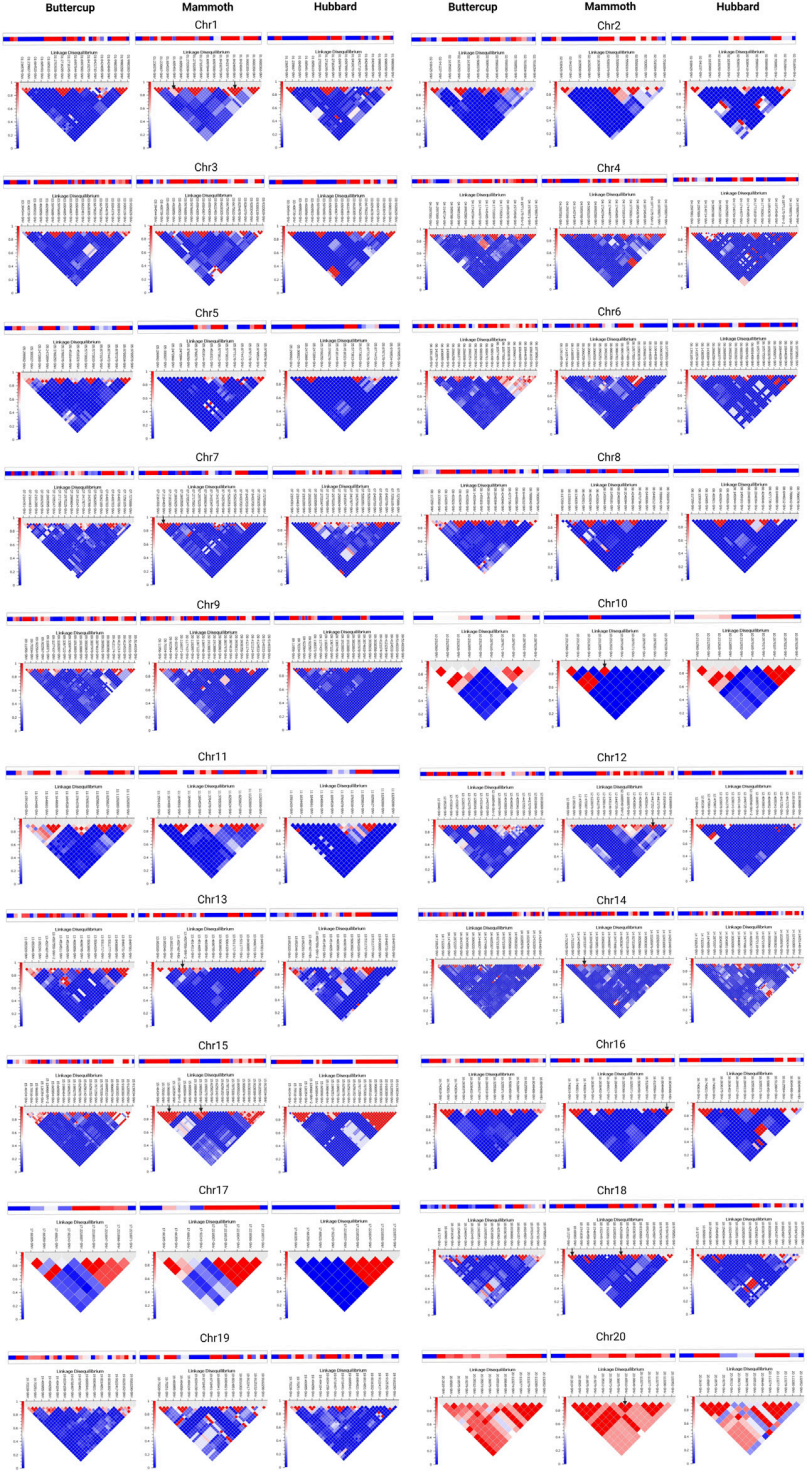
Distribution of haplotypes shared across buttercup, mammoth, and hubbard groups for chromosomes 1 to 20.

Study of haplotype-sharing is more informative than single-variant approaches for inferring demographic history at the population level without requiring deep whole-genome sequencing. We computed haplotype blocks via the LD interface by using the method developed by (Gabriel et al., 2002). This algorithm produced haplotype blocks that appear as black outlined pentagons at the top of the chromosome-specific LD plot (Figures 4A,B). Chromosome-wise distribution of haplotypes for buttercup (left), mammoth (center) and hubbard (right) for chro-1 to 10 in Figure 4A and for chro-10 to 20 in Figure 4B suggests sharing of ancestral haplotypes, decay of ancestral (buttercup or hubbard) haplotypes and formation of new haplotypes in the mammoth group. This helps to understand the recent effective population-size changes in the mammoth group and continuous gene flow and directional selection for fruit size. Lastly, we showed that ancestral haplotype sharing was enriched or decayed, with the formation of new haplotypes by accumulation of private alleles. Our work provides a general framework for haplotype sharing among the horticulture groups of *C. maxima* to gain insight into the evolutionary origins of rare variants contributing to the giant fruit size and associated changes.

## Association mapping

**FIGURE 5:**
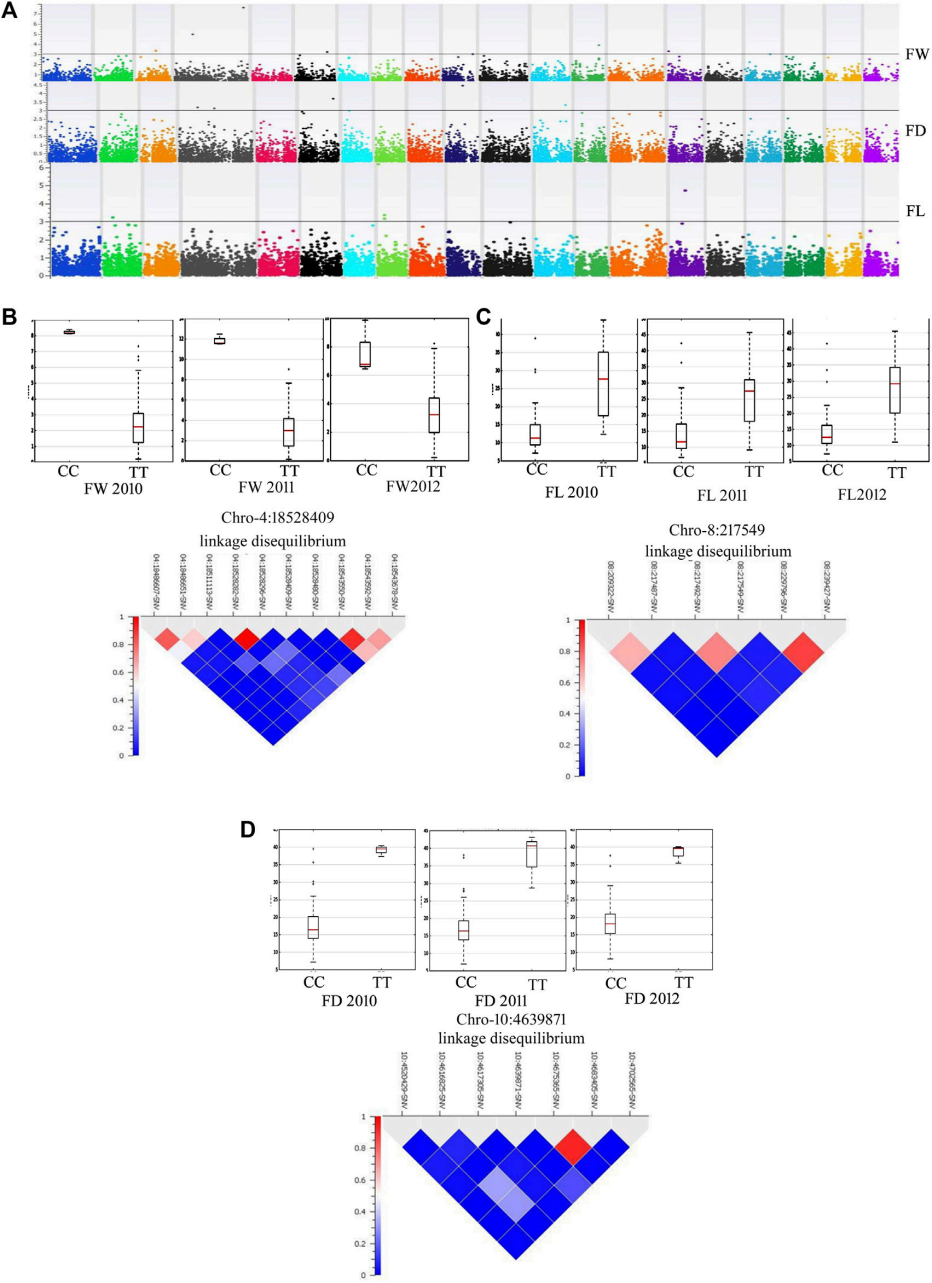
**(A)** Manhattan plots showing GWAS results for various fruit traits [FW: fruit weight (lb), FL: fruit length (cm) and FD: fruit diameter (cm)] across the years 2010, 2011 and 2012. **(B)** Allelic effects of SNP 18528409 on chromosome 4 for FW. **(C)** Allelic effects of SNP 217549 on chromosome 8 for FL. **(D)** Allelic effect of SNP 4639871 on chromosome 10 for FD.

By adopting multiple-locus mixed linear models developed by the EMMAX method with a PC matrix and identity-by-descent indices as covariates for FW, FL, FD, RDL and SOL, GWAS produced a robust set of associated SNPs (Supplementary Table S6; Figures 5A). Most associated SNPs were in the candidate genes and the strength of association was tested with an FDR test. Allelic effects were compared across the three independent seasons (Figures 5B–D). Our GWAS resolved important loci on chromosomes 4, 5, 6, 12, 13, 15, and 17 for fruit weight. A missense variant in the homeobox-leucine zipper protein ATHB-20-like (S04_18528409) was highly significantly associated (FDR = 0.000004) with fruit weight in the mammoth type and the allelic effect was consistent across the 3 years (Figure 5B). A cofactor (S08_217549) on chromosome 8 was detected in strong association with fruit length, showing a consistent allelic effect for this trait across the 3 years (Figure 5C). A missense variant (S10_4639871) on the translocation protein SEC62 was a cofactor for fruit diameter and showed a high allelic effect across the 3 years (Figure 5D).

## Common SNPs and genes

Some of the positive selection genes (PSGs) were also found in strong association with various traits in this GWAS. S01_644006, a synonymous variant on methyl-CpG-binding domain-containing protein 11-like, showed a strong association with both FL and RDL across the years and was a PSG. Pentatricopeptide repeat-containing protein was in significant association with FW, FL and FD and RDL. Pentatricopeptide repeat-containing protein was a major gene in this study and a high positive selection gene. Leu-rich repeat (LRR) receptor-like serine/threonine-protein kinase was a PSG and in the GWAS, manifested a strong association with FW, FL, and FD. E3 ubiquitin-protein ligase COP1-like was also a PSG and was found in association with RDL; likewise, WD repeat-containing protein, a PSG, was in association with FW and played a role in genetic differentiation between hubbard and mammoth groups.

## Discussion

The mammoth group is a showy type of squash (also named Display or Giant). It is characterized by its giant globular mature fruits, and has long been known to produce the largest fruits in the plant kingdom (López-Anido, 2021). Interest in large pumpkins derives from exhibits in agricultural fairs in North American or European rural life for the last 150 years (Janick, 2008). The mammoth type fruits have orange, yellow, cream and/or white color, while the flesh is light orange, yellow or cream color. The flesh has 4.3–5.1% dry matter, while the seed length to width ratio is >1.8, the largest of all *C. maxima* cultivar groups (López-Anido, 2021). The current admixture analysis showed that the mammoth group largely evolved from the hubbard type, known as Hubbardiana. The hubbard fruit is characterized by its elliptical-acorn or ovate shape, tapering curved to one or both ends, of medium to large size (2–6 kg) with uneven to warted type rind (López-Anido, 2021). The hubbard rind color varies from green, blue-gray, orange to cream-white, with or without longitudinal stripes. Plants in hubbard group show a viny growth habit; some new cultivars may be intermediate in internode length, as for a semi-bush type, and very few show mottled leaves similar to the plant mammoth type (Castetter, 1925; von Grebenščikov, 1958). Pan et al. (2021) compared Atlanta giant, a mammoth type, with hubbard and noted increased fruit cell number and massive cell volume at harvest stage, accompanied by larger leaves, larger peduncle vascular cross area and higher phloem sap sugar concentration. Cucurbit fruit size is essentially controlled by three processes in the ovary: cell differentiation (e.g., the definition of the number of carpels), cell division and cell expansion (Colle et al., 2017).

Some of the PSGs revealed in this study that have direct roles in hormonal regulation were auxin efflux carrier component, auxin-responsive protein SAUR21-like, auxin-responsive protein, SAM domain-containing protein, S-acyltransferase, 1-aminocyclopropane-1-carboxylate synthase, ethylene-responsive transcription factor 2-like and hydroxyacyl-CoA dehydrogenase. Among the PSGs involved in CLV-WUS signaling were cell division protein ftsZ, cell number regulator 6-like, trypsin family protein, DUF641 domain-containing protein, pectin lyase-like superfamily protein, and WD-40 repeat-containing protein. SEC62 was a cofactor in GWAS, and homeobox-leucine zipper protein ATHB-20-like (S04_18528409) was detected to be in strong association. MYB36-like is a MADS-box family transcription factor. The largest allelic effect in this GWAS for fruit size was of LRR receptor-like kinase (RLK), consistently shown across the years. This gene was also major marker for fruit size increase in a high-resolution genetic mapping of a biparental cross involving Atlanta giant and hubbard (Pan et al., 2022). In a yeast two-hybrid assay in strawberry, RLK showed an interaction with a putative ABA receptor, which in turn induced ABA and ethylene (Hou et al., 2017). Although the structural features of LRR-RLKs are similar, their ligand molecules vary from steroids (brassinolides) to peptides. CLAVATA 3 (CLV3), a secreted protein, is recognized by its receptor CLV1 to maintain the homeostasis of the apical meristem. RLKs are also involved in a number of developmental processes such as upregulating BRASSINOSTEROID INSENSITIVE 1 (BRI1), a major receptor that mediates cell elongation required for growth and development of the plant (Pesaresi et al., 2014). Endoreduplication is another phenomenon that influences fruit growth rate at the level of the cell expansion rate in fleshy fruits that develop rapidly (in <13 weeks), consisting of three to eight rounds of endocycle, in all Solanaceae and Cucurbitaceous species (Chevalier et al., 2011). The progression within the distinct phases of the plant endocycle requires the activity of a class of conserved heterodimeric protein complexes consisting of a catalytic subunit called the CDK–CYC subunits. Expression analyses in previous studies (Sun et al., 1999; Gonzalez et al., 2007) revealed that the transcripts for *WEE1*, a kinase family containing three serine/threonine motif, contribute to the endoreduplication process (Ghelli Luserna Di Rorà et al., 2020). Serine/threonine kinases were a major PSG and GWAS hit in the current analysis.

We also identified that genes in the ubiquitin-proteasome pathway—ubiquitin carboxyl-terminal hydrolase, RING-type E3 ubiquitin transferase, E3 ubiquitin-protein ligase COP1-like, cucumisin-like, serine/threonine phosphatase, RING-H2 finger protein ATL34-like—were associated with fruit size and have catalytic roles. The completion of mitosis and progression from mitosis back into interphase requires the loss of CDK–CYC complex activity, which occurs via proteolytic destruction of the cyclin moiety by a specific E3-type ubiquitin ligase and other proteases (Heyman and De Veylder, 2012).

In cucurbits, 1-aminocyclopropane-1-carboxylate synthase and CLAVATA3 loci are known to have pleiotropic effects on fruit size/shape (Pan et al., 2020). *FW2.2*, a validated tomato fruit size gene that fixes the protein to the plasmalemma via its transmembrane-spanning domains contains a PLAC8 motif with two conserved cysteine-rich domains separated by a variable region predicted to play a role across the transmembrane segments (Guo et al., 2010). In this study, a missense mutation in cysteine-rich receptor-like protein kinase 42 on chro-14 was highly associated with fruit length, contributing a phenotypic variance of 15%. FW 3.2 is another tomato fruit size/weight gene that encodes a P450 enzyme of the CYP78A subfamily, previously known as KLUH, which also appeared in this analysis associated with mammoth fruit formation (Anastasiou et al., 2007; Chakrabarti et al., 2013).

Among the other common genes across positive selection genes and GWAS across the years, we noted methyl-CpG-binding domain (MBD) proteins that play important roles in epigenetic gene regulation and have diverse molecular, cellular, and biological functions in plants (Qu et al., 2021). In addition, we noted that pentatricopeptiderepeat (PPR) families were in strong association with various fruit size traits. PPRs are nucleus-encoded post-transcriptional regulators that bind to specific chloroplast mRNAs and control their maturation and/or stabilization by acting as adaptors. Through these processes, PPRs may mediate subtle regulatory changes such as the assembly and abundance of specific protein complexes in response to developmental stimuli (Pesaresi et al., 2014).

## Conclusion

We have critically analyzed the formation of the horticulture group mammoth by using a set of genomic and population genetic analyses. To track the genetic signatures of the mammoth group evolution, we studied admixture, chromosome-wise nucleotide divergence, genome-wide haplotype sharing and population demography of various groups of *C. maxima*. We assembled a toolkit of genes related to hormonal regulation by scanning private alleles for mammoth type, selection genes across the chromosomes and GWAS for fruit size traits across three consecutive years. Intriguingly, we identified a set of common genes with known functions in fruit development and also genes that repeatedly appear in various population genetic analyses in this research. The current study reiterates that the increase in fruit size involves shifts in the regulation of cell division and cell expansion. Several molecular mechanisms are involved in the determination of fruit size, including hormonal regulation, CLV-WUS signaling pathway, MADS-box family, and ubiquitin-proteasome pathway. Chromosome-wise distribution of haplotypes for buttercup, mammoth and hubbard in a suggested sharing of ancestral haplotypes (buttercup or hubbard); decay of ancestral haplotypes; and formation of new haplotypes by the accumulation of private alleles might be the evolutionary force behind the formation of the mammoth group. This study helps to understand genomic-level changes occurring during breeding and directional selection for giant-sized fruits. Our work provides a general framework for genome-wide divergence, population differentiation and admixture among the horticulture groups of *C. maxima* and provides insight into the evolutionary origins of rare variants contributing to the giant fruit size and associated changes.

## Data Availability

The datasets presented in this study can be found in online repositories. The names of the repository/repositories and accession number(s) can be found below: Sequence Read Archive (SRA) at NCBI under the Bio Project accession number PRJNA870945.

## Supplementary material

Supplementary Figure S1 Fruits of various horticulture groups: 1. Australian 2. mammoth 3. Buttercup 4. Turban 5. Zapallito 6. Banana, and 7. Hubbard.

Supplementary Figure S2 Box plots for various representative groups (I: Australian, II: Buttercup, Zapallito, Turban, III: Hubbard and Banana and IV: Mammoth) for fruit weight (lbs), fruit length (cm), fruit diameter (cm), ratio of fruit length and diameter and soluble solids (%).

Supplementary Figure S3 Principal component analysis chart was made using first two eigen vectors involving all the accessions belonging to various groups.

Supplementary Figure S4 2-kb sliding windows of chromosomes 2, 3, 5, 6, 7, 9, 10, 11, 13, 14, 15, 17, 18, and 19 depicting pairwise *F*_*ST*_s of various groups (I: Australian, II: Buttercup, Zapallito, Turban, III: Hubbard and Banana and IV: Mammoth).

Supplementary Figure S5 Significant positive and negative Tajima’s D values for the mammoth group across various chromosomes.

Supplementary Figure S6 Gene ontologies (GOs) for various genes underlying positive and negative Tajima’s D indices.

Supplementary Figure S7 Box plots of genomewide Tajima’s D for groups- I: Australian, II: Buttercup, Zapallito, Turban, III: Hubbard and Banana and IV: Mammoth in the study.

Supplementary Table S1 List of accessions.

Supplementary Table S2 Fruit trait variation across the years for various horticulture groups in the study.

Supplementary Table S3 Gene ontologies for genes with highly positive Tajima’s D.

Supplementary Table S4 Gene ontologies for genes with highly negative Tajima’s D.

Supplementary Table S5 Overall Tajima’s D values for entire set of accessions in this study.

Supplementary Table S6 Association statistics of single nucleotide polymorphisms significantly associated with various fruit size traits by Efficient Mixed-Model Association eXpedited (EMMAX).
