# Supplementary figures and images for: What makes a giant fruit? Assembling a genomic toolkit underlying various fruit traits of the mammoth group of Cucurbita maxima
Source: Front Genet. 2022 Sep 20;13:1005158. doi: 10.3389/fgene.2022.1005158 (PMC9531317; doi:10.3389/fgene.2022.1005158)

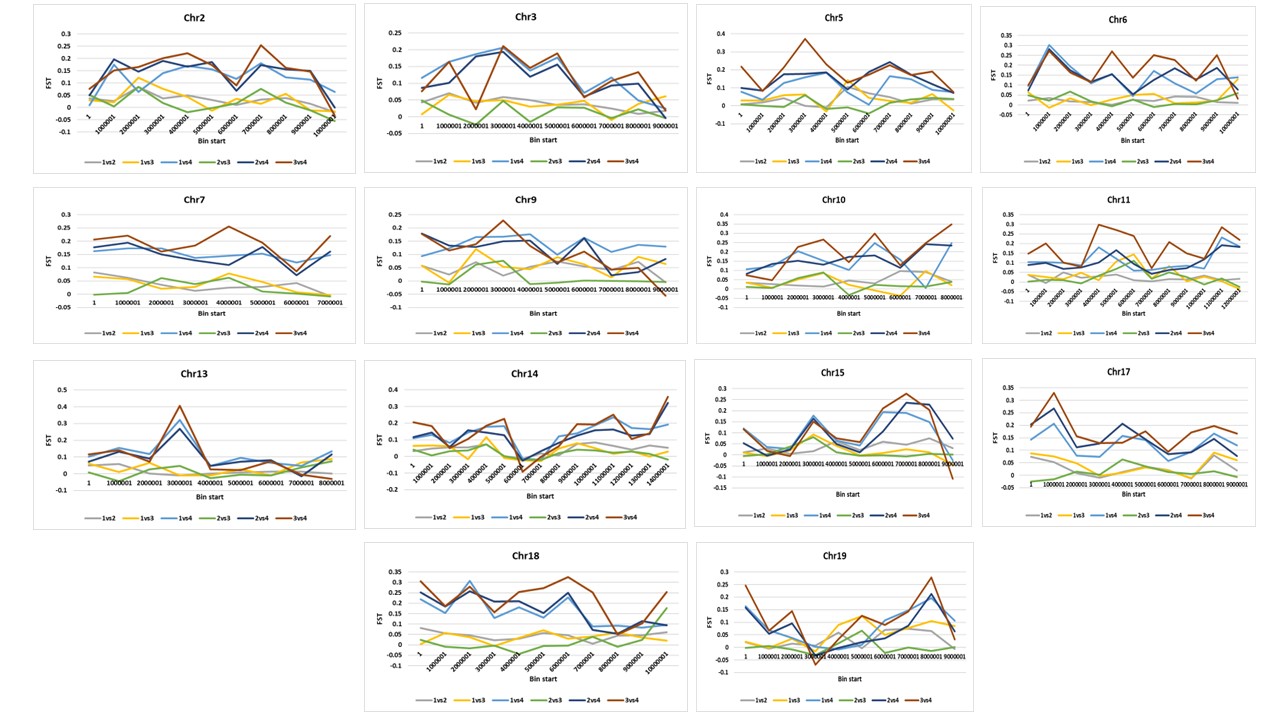

Supplement: Supplementary file 5 [file Image4.JPEG]

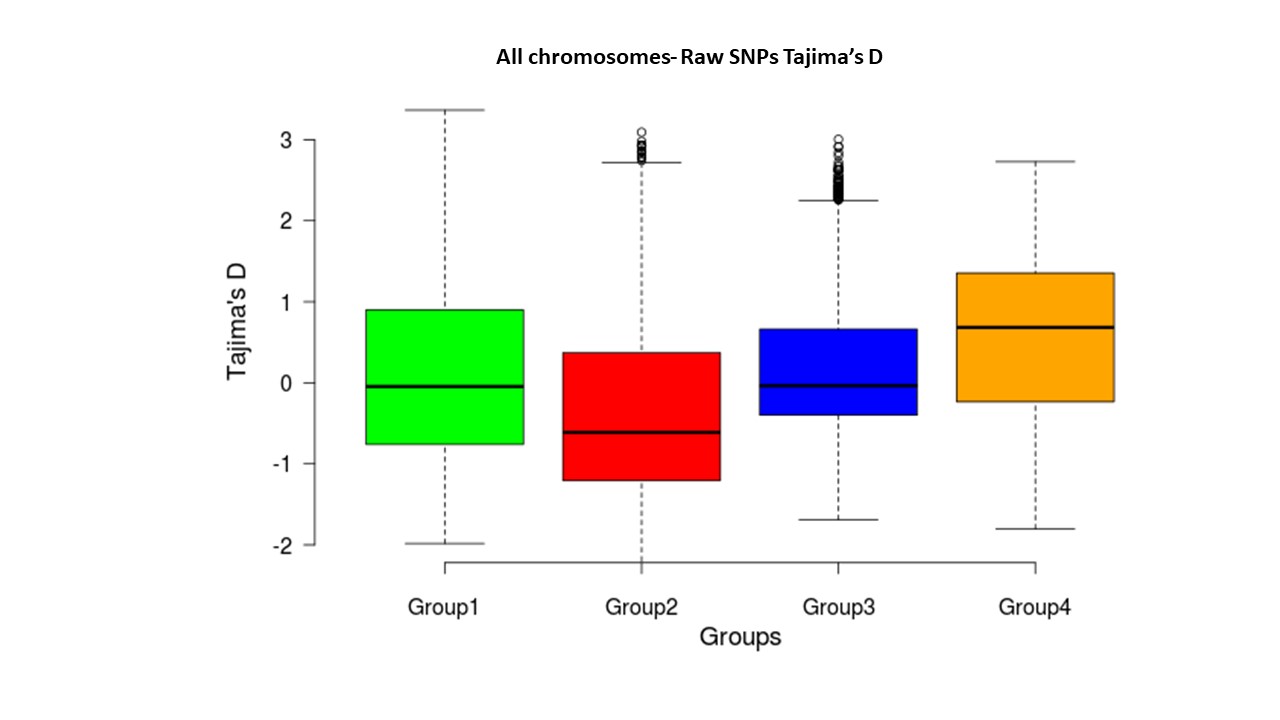

Supplement: Supplementary file 6 [file Image7.JPEG]
