# Supplementary material for: What makes a giant fruit? Assembling a genomic toolkit underlying various fruit traits of the mammoth group of Cucurbita maxima
Source: Front Genet. 2022 Sep 20;13:1005158. doi: 10.3389/fgene.2022.1005158 (PMC9531317; doi:10.3389/fgene.2022.1005158)
Supplement: Supplementary file 11 [file Table6.DOCX]

| Chromosome | SNP | P-Value (After Step 2) | #NAME? | Predictor Beta | FDR | Prop. Var. Explained | Manhattan Category (After Step 2) | Minor Allele Frequency | Gene ID | Gene Region (Combined) | MA→MI | **Variant type** | **Annotation** | |
| --- | --- | --- | --- | --- | --- | --- | --- | --- | --- | --- | --- | --- | --- | --- |
| **FRUIT WEIGHT** | | | | | | | | | | | | | | |
| 6 | S06_8594924 | 0.000632 | 3.1 | 1 |  | 0.156722 | Chr 06 | 0.080882 | CmaCh06G012950 | Exon | T→G | synonymous_variant | | Pentatricopeptide repeat-containing protein |
| 13 | S13_6969693 | 0.000137 | 3.8 | 1.1 |  | 0.191293 | Chr 13 | 0.109375 | CmaCh13G008360 | UTR5 | A→G | 5_prime_UTR_variant | | LRR receptor-like serine/threonine-protein kinase GSO1 |
| 15 | S15_376767 | 0.000539 | 3.2 | 1.5 |  | 0.160385 | Chr 15 | 0.043478 | CmaCh15G000800 | Exon | G→A | missense_variant | | Chloride channel protein |
| 17 | S17_6755470 | 0.000976 | 3 | 0.8 |  | 0.146747 | Chr 17 | 0.150943 | CmaCh17G008880 | Intron | A→G | splice_region_variant&intron_variant | | Protein kinase superfamily protein |
| 4 | S04_18528409 | 3.30E-10 | 9.4 | 3.6 | 4.26E-06 | ? | Cofactor | 0.058333 | CmaCh04G027560 | Exon | T→C | missense_variant | | homeobox-leucine zipper protein ATHB-20-like |
| 12 | S12_6623668 | 0.000996 | 3 | -0.75 |  | 0.146282 | Chr 12 | 0.365672 | CmaCh12G008780 | Exon | A→G | missense_variant | | SET domain-containing protein |
| 5 | S05_1245159 | 0.000105 | 3.9 | 1.4 |  | ? | Cofactor | 0.198276 | CmaCh05G002880 | Exon | C→A | missense_variant | | lysine-specific histone demethylase 1 homolog 3-like |
| 7 | S07_2969566 | 0.000138 | 3.8 | -1 |  | 0.191111 | Chr 07 | 0.351563 | CmaCh07G006770 | Exon | A→G | frameshift_variant | | WD40 repeat-containing protein HOS15 |
| **FRUIT LENGTH** | | | | | | | | | | | | | | |
| 8 | S08_217549 | 6.49E-07 | 6.1 | 6.8 | 0.008389 | ? | Cofactor | 0.783784 | CmaCh08G000410 | Exon | C→T | synonymous_variant | | Unknown protein |
| 2 | S02_2557332 | 0.000629 | 3.2 | -3.4 |  | 0.158982 | Chr 02 | 0.447368 | CmaCh02G004850 | Exon | C→T | missense_variant | | benzyl alcohol O-benzoyltransferase-like |
| 1 | S01_644006 | 0.000351 | 3.4 | -4.4 |  | 0.172401 | Chr 01 | 0.485915 | CmaCh01G001430 | Exon | C→T | synonymous_variant | | methyl-CpG-binding domain-containing protein 11-like |
| 4 | S04_4774854 | 0.000422 | 3.3 | -4.2 |  | 0.168167 | Chr 04 | 0.378571 | CmaCh04G009250 | Exon | C→A | synonymous_variant | | organic cation/carnitine transporter 4 |
| 6 | S06_8949976 | 0.000777 | 3.1 | 8.2 |  | 0.154084 | Chr 06 | 0.070313 | CmaCh06G013700 | Intron | T→A | downstream_gene_variant | | 1,2-dihydroxy-3-keto-5-methylthiopentene dioxygenase |
| 11 | S11_2804463 | 3.92E-05 | 4.4 | 9.2 |  | ? | Cofactor | 0.088235 | CmaCh11G005830 | Exon | T→C | synonymous_variant | | Pentatricopeptide repeat-containing protein |
| 14 | S14_3325547 | 0.000705 | 3.1 | 5.7 |  | 0.15632 | Chr 14 | 0.139344 | CmaCh14G006630 | Exon | A→G | missense_variant&splice_region_variant | | cysteine-rich receptor-like protein kinase 42 |
| 16 | S16_1164588 | 0.000326 | 3.4 | -4.4 |  | 0.174084 | Chr 16 | 0.292857 | CmaCh16G002500 | Exon | C→G | synonymous_variant | | Leucine-rich repeat protein kinase family protein, putative |
| 6 | S06_9203848 | 0.000205 | 3.6 | -3.6 |  | ? | Cofactor | 0.330508 | ? | intergenic | C→T | downstream_gene_variant | | basic helix-loop-helix (bHLH) DNA-binding superfamily protein |
| 10 | S10_4639871 | 3.51E-05 | 4.4 | 7.3 |  | ? | Cofactor | 0.070175 | CmaCh10G009270 | Exon | C→T | missense_variant | | Translocation protein SEC62 |
| 12 | S12_8591840 | 0.000485 | 3.3 | 3 |  | 0.165008 | Chr 12 | 0.421053 | CmaCh12G010880 | Exon | C→T | missense_variant | | Receptor-like serine/threonine-protein kinase |
| **FRUIT DIAMETER** | | | | | | | | | | | | | | |
| 5 | S05_2022266 | 8.64E-06 | 5 | -4.3 | 0.055829 | ? | Cofactor | 0.454545 | CmaCh05G004410 | Intron | A→C | intron_variant | | Beta-glucosidase |
| 6 | S06_8594924 | 0.000619 | 3.2 | 4.7 |  | 0.159352 | Chr 06 | 0.08209 | CmaCh06G012950 | Exon | T→G | synonymous_variant | | Pentatricopeptide repeat-containing protein |
| 7 | S07_4032025 | 0.000946 | 3 | 2.7 |  | 0.14949 | Chr 07 | 0.453125 | CmaCh07G008640 | Exon | G→A | missense_variant | | serine/threonine-protein kinase D6PKL1 |
| 11 | S11_2685776 | 0.000542 | 3.2 | 3.2 |  | 0.162407 | Chr 11 | 0.235294 | CmaCh11G005550 | Intron | T→C | downstream_gene_variant | | GTP-binding nuclear protein |
| 11 | S11_4535309 | 0.000906 | 3 | -2.5 |  | 0.150492 | Chr 11 | 0.481818 | CmaCh11G008890 | UTR3 | A→G | 3_prime_UTR_variant | | pentatricopeptide repeat-containing protein At1g62350 |
| 10 | S10_4639871 | 8.6E-05 | 4 | 6.7 |  | ? | Cofactor | 0.070175 | CmaCh10G009270 | Exon | C→T | missense_variant | | Translocation protein SEC62 |
| 15 | S15_3650814 | 0.000652 | 3.1 | -2.9 |  | 0.158145 | Chr 15 | 0.308333 | CmaCh15G007480 | Intron | A→G | intron_variant | | Ty3-gypsy retrotransposon protein |
| **RATIO OF DIAMETER AND LENGTH** | | | | | | | | | | | | | | |
| 1 | S01_644006 | 6E-06 | 5.2 | -0.4 | 0.038757 | ? | Cofactor | 0.485915 | CmaCh01G001430 | exon | C→T | synonymous_variant | | methyl-CpG-binding domain-containing protein 11-like |
| 1 | S01_4000830 | 0.000592 | 3.2 | 0.4 |  | 0.160392 | Chr 01 | 0.066038 | CmaCh01G007460 | intron | A→G | downstream_gene_variant | | Protein-serine/threonine phosphatase |
| 2 | S02_7403015 | 0.000804 | 3 | 0.3 |  | 0.153275 | Chr 02 | 0.106061 | CmaCh02G012620 | exon | A→G | synonymous_variant | | TPR repeat-containing thioredoxin TTL2-like |
| 4 | S04_6258020 | 0.000666 | 3.1 | 0.4 |  | 0.157642 | Chr 04 | 0.087719 | CmaCh04G012270 | exon | G→T | missense_variant | | serine/arginine-rich splicing factor RS2Z32-like isoform X1 |
| 5 | S05_9317523 | 5.61E-06 | 5.2 | -0.3 | 0.072522 | ? | Cofactor | 0.422535 | CmaCh05G012090 | exon | T→C | synonymous_variant | | E3 ubiquitin protein ligase DRIP2-like isoform X1 |
| 9 | S09_4781025 | 0.000448 | 3.3 | 0.4 |  | 0.166811 | Chr 09 | 0.066038 | CmaCh09G009510 | exon | T→G | synonymous_variant | | Rho GTPase-activating protein |
| 17 | S17_2724131 | 0.00016 | 3.7 | 0.4 |  | 0.19036 | Chr 17 | 0.094828 | ? | intergenic | T→A | intergenic_region | | 1-aminocyclopropane-1-carboxylate synthase 1 |
| 19 | S19_5042656 | 0.000735 | 3.1 | -0.3 |  | ? | Cofactor | 0.450704 | CmaCh19G004180 | exon | T→C | synonymous_variant | | remorin-like |
| 4 | S04_18895265 | 0.000448 | 3.3 | -0.3 |  | ? | Cofactor | 0.291045 | CmaCh04G028250 | intron | C→T | downstream_gene_variant | | Pentatricopeptide repeat-containing protein |
